# Robust Camera-Based Eye-Tracking Method Allowing Head Movements and Its Application in User Experience Research

**DOI:** 10.3390/jemr18060071

**Published:** 2025-12-01

**Authors:** He Zhang, Lu Yin

**Affiliations:** 1 School of Art and Design, Wuhan University of Technology, Wuhan 430070, China; 2 School of Design, Hunan University, Changsha 410082, China

**Keywords:** user experience analysis, camera-based eye-tracking, head movement, head-pointing, gaze point estimation

## Abstract

Eye-tracking for user experience analysis has traditionally relied on dedicated hardware, which is often costly and imposes restrictive operating conditions. As an alternative, solutions utilizing ordinary webcams have attracted significant interest due to their affordability and ease of use. However, a major limitation persists in these vision-based methods: sensitivity to head movements. Therefore, users are often required to maintain a rigid head position, leading to discomfort and potentially skewed results. To address this challenge, this paper proposes a robust eye-tracking methodology designed to accommodate head motion. Our core technique involves mapping the displacement of the pupil center from a dynamically updated reference point to estimate the gaze point. When head movement is detected, the system recalculates the head-pointing coordinate using estimated head pose and user-to-screen distance. This new head position and the corresponding pupil center are then established as the fresh benchmark for subsequent gaze point estimation, creating a continuous and adaptive correction loop. We conducted accuracy tests with 22 participants. The results demonstrate that our method surpasses the performance of many current methods, achieving mean gaze errors of 1.13 and 1.37 degrees in two testing modes. Further validation in a smooth pursuit task confirmed its efficacy in dynamic scenarios. Finally, we applied the method in a real-world gaming context, successfully extracting fixation counts and gaze heatmaps to analyze visual behavior and UX across different game modes, thereby verifying its practical utility.

## 1. Introduction

Eye-tracking data can reflect visual attention [1,2] and provides reliable explanations for user experience [3]. By employing eye-tracking technology, researchers can determine where users are looking at a given moment and their scan sequence, thereby aiding in the understanding of how users process visual information. Generally, users need to wear special hardware devices, such as eye trackers or VR helmets, to realize gaze tracking, which limits the application of eye tracking in practice scenarios. Additionally, these high-precision devices are relatively expensive, which could exceed the cost that the ordinary person can afford [4], and continuously wearing them may cause eye or head discomfort [5].

Recent studies have introduced camera-based methods to capture eye features and estimate the gaze point. These methods do not require additional hardware equipment except a computer with a webcam. Although the accuracy and stability of camera-based methods still have a significant gap compared to specialized eye-tracking devices, they are more acceptable due to their lower price and convenience. Some scholars have also chosen the camera-based eye-tracking method instead of the eye-tracker to analyze user behavior [6,7]. In addition to accuracy and robustness, these methods still face the critical limitation of being sensitive to head motion [8]. If the user’s head moves, the accuracy of predicting the gaze point could plummet. Therefore, most studies require users to keep their heads as fixed as possible. For short periods, this is not a problem. However, if the duration grows, the requirement for head fixation is unfriendly and unnatural since the user will instinctively move the head when gazing at different positions [9].

To address the above problems, we propose a camera-based eye-tracking method allowing natural head movements. The workflow of our method is as follows: the user first gazes at five points on the screen to perform calibration, including one screen center point and four edge points. Among them, the center point is used to obtain the benchmarks of the pupil center, eye corner, and head direction detected by the facial landmark detection module in MediaPipe [10]. The edge points help confirm the related scale coefficients for gaze point prediction. After calibration, the eye-tracking process starts. The first step is determining whether the head moves based on the difference between the current head pose and the head pose benchmark. If yes, our method will further compute the head-pointing coordinates and update the benchmark features. Subsequently, the gaze point coordinates will be obtained by multiplying the difference vector between the current pupil center and the updated benchmark by the scale coefficients. To test the accuracy of the proposed eye-tracking method, we recruited 22 individuals to participate in the experiment. Each individual needed to finish two tasks using our method under head fixation and head movement to gaze at 16 fixation points and a moving target. The error between the prediction gaze points and the actual test points is used to assess the accuracy. Additionally, we also apply our method in actual gaming scenarios. We effectively analyzed users’ visual gaze behavior and experience across different game modes by extracting the fixation count and gaze heatmaps, thereby validating the effectiveness of this method.

In sum, the main contributions are as follows:

- We proposed a robust camera-based eye-tracking method that allows head movements.
- A fixation task was performed to test the accuracy. The results showed that our method outperforms most existing methods under head fixation and head motion.
- A smooth pursuit task was performed to verify the performance of our method when gazing at a moving target.
- We also conducted a case study to demonstrate the effectiveness of our method in user experience research.

## 2. Related Work

### 2.1. User Experience Analysis by Eye-Tracking

Some studies have employed eye-tracking for digital product usability evaluation, including apps [11,12] and websites [3,13,14,15,16,17]. For instance, Liu et al. [11] investigated how the color and border style of mobile app icons influence user search efficiency and overall experience. The authors utilized eye-tracking metrics such as fixation duration and fixation count to evaluate search efficiency, while assessing user experience through satisfaction and usability. Results indicated that icons with different colors and rounded square borders contributed to improved search efficiency and user experience. Naeini et al. [3] examined how eye-tracking metrics correlate with usability across different shopping websites. Experimental results revealed a strong negative correlation between metrics such as fixations, saccades, and saccade paths and usability. Specifically, websites with lower usability scores exhibited higher visual complexity in their interfaces, more saccades, and longer saccade paths. Beyond apps and websites, He et al. [18] employed eye-tracking to investigate how different design features of electronic navigation screens in urban rail vehicles influence users’ visual behavior and satisfaction. Guo et al. [19] investigated the effect of mobile news interface complexity on user satisfaction. Results indicated that eye-tracking metrics collected under different complexities can help explain user satisfaction.

There are also some studies that utilized eye-tracking technology to support the analysis of user emotions and experiences. For instance, Guo et al. [20] employed eye-tracking to explore users’ real-time responses to smartphone images during browsing and goal-directed tasks. Findings revealed that users were more easily drawn to smartphone images with high user experience in the browsing task, and images with low user experience elicited greater pupil dilation. In the goal-directed task, smartphone images with high user experience received longer viewing durations. Cybulski et al. [21] investigated differences in user experience when interacting with various web maps. The number of fixations, first fixation, and saccade amplitude were employed to evaluate interaction experiences. Results indicate that different arrangements of identical interactive tools may produce varying effects on users’ visual experience and efficiency when interacting with maps.

Eye-tracking technology has also found extensive application in the gaming field. For instance, Jiang et al. [22] employed eye-tracking and subjective interaction experience scales to investigate players’ interaction experiences across two game tasks. The study revealed that distinct eye-tracking metrics can effectively reflect and predict interaction experiences under different task conditions. Lan et al. [23] explored how players process written language during video game play using eye-tracking technology. Krebs et al. [24] tested and validated the practicality of applying eye-tracking to analyze users’ cognitive performance in puzzle games. Additionally, Wang et al. [25] investigated the eye-tracking characteristics of players at different skill levels, including average number of fixations, average fixation duration, and gaze trajectories. The study revealed that professional players exhibited more fixations and more efficient visual search strategies compared to non-professional players.

In summary, the majority of studies primarily employed eye-tracking devices to measure eye-movement data. This method has become a crucial tool in user experience research due to its ability to capture users’ visual attention. Researchers can gain deep insights into users’ visual search processes and attention allocation during interactions with products and interfaces by analyzing key metrics such as pupil size, fixation count, gaze trajectories, and heatmaps. These data play a crucial role in evaluating user emotion, experience, usability, and satisfaction. However, some limitations still exist. For instance, eye-tracking devices are relatively expensive, particularly high-precision eye trackers, which carry significant purchase and maintenance costs. Additionally, some eye-tracking equipment requires users to wear devices on their heads, such as head-mounted or eyeglass-style trackers, which may inconvenience users with myopia (especially those who already wear glasses), potentially disrupting natural behavior, causing discomfort, and restricting head movement.

### 2.2. Camera-Based Eye-Tracking Methods

Eye-tracking methods are mainly divided into two categories: model-based and appearance feature-based [4,9]. The former usually consists of a 3D eye model, which is fitted by extracting features such as pupil center [26,27], iris contour [28], and corneal infrared reflections [29,30] to estimate gaze. These approaches usually require one or multiple infrared light sources to extract features, thus constraining their practical applications. With the continuous development of computer vision, it has become easier to capture eye-related features using visible cameras. Researchers have been dedicated to developing appearance-based eye-tracking approaches in recent years. For example, Papoutsaki et al. [31] trained a ridge regression model to map the multidimensional appearance feature vectors of the eye region image to the screen coordinates by multipoint calibration. Blignaut [32] estimated gaze points using the pupil-glint vector and conducted experiments to investigate the effect of different numbers of calibration points on the accuracy. The results indicated that prediction accuracy could improve when the number exceeded 14. However, excessive points can lead to a longer calibration time. Some studies [33,34] reduced the number of calibration points by simplifying the projection model. One of the commonalities of the above methods is that they all establish the relationship between different features and screen coordinates through calibration. However, these methods require the subject to keep the head fixed because they are sensitive to head motions.

Early methods of compensating for head motion [29,35,36] typically required multiple cameras to estimate the 3D position of the eyes, followed by mathematical transformations to compute the gaze direction. Although they were able to handle head movements to some extent, the use of multiple devices led to increased costs and greater complexity and limited the application in practice [37]. In recent years, some studies have also focused on this issue and introduced powerful CNN to address it. For example, Sj et al. [38] used a dual-stream convolutional autoencoder network structure to extract the features of the left and right eyes and regress the gaze point coordinates. To minimize the effect of head motion, the authors integrated the head pose into the eye feature vectors and proposed roll angle correction and vertical gaze compensation methods. The reported mean pixel errors range from 22 to 200 pixels. Falch et al. [39] built the projection relationship between gaze direction and screen gaze point via a 4-point calibration. In addition, the authors suggested utilizing structure from motion to track user movements and compensate for horizontal head motions. The results demonstrate the effectiveness of this method in addressing large head movements. Liu et al. [40] presented an improved cross-ratio-based gaze estimation method, which used two infrared light sources and the 3D eyeball imaging model to estimate the 3D corneal center and then determined the pupil reference plane to guarantee coplanarity for subsequent gaze point estimation. The method obtains a satisfactory accuracy of 1.33 degrees under free-head movement. Banks et al. [41] proposed a head motion compensation method based on eye corner displacement. Specifically, the authors first estimated the initial gaze point coordinates by mapping pupil-glint vectors. Next, they employed a eye detector pre-trained on their manually labeled eye dataset to detect the eye regions and a derivative-based corner detector to locate the eye corners. The offset of the eye corner relative to the calibrated position was then fed into a modified regression model to calculate the gaze point error and compensate for the gaze point. The results showed that the accuracy of this method is better than the existing methods. The weakness is that the calibration time was relatively high (close to 90 s).

Although numerous methods have studied the head motion problem, some limitations remain unresolved, such as low accuracy, long calibration time, additional equipment required, and inability to handle significant head movements. Unlike the above methods, this paper enhances the performance under head motions by continually detecting the head state and updating the benchmark features after head movements.

## 3. Methodology

**Figure 1:**
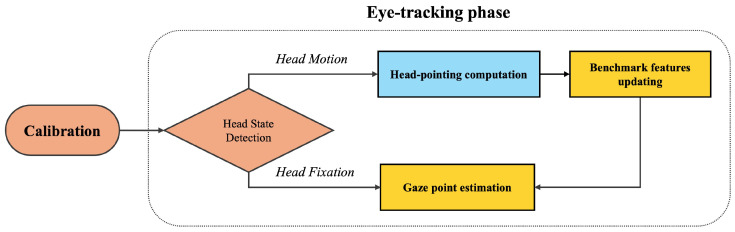
Framework of the proposed eye-tracking method.

This paper develops a camera-based eye-tracking method allowing head motions. Figure 1 shows the framework, which involves two phases: calibration and eye tracking. The former phase aims to obtain the benchmark features and gaze point prediction-related coefficients. The latter contains several sub-modules: head-state detection, head-pointing computation, benchmark features updating, and gaze point prediction.

### 3.1. Features Extraction

**Figure 2:**
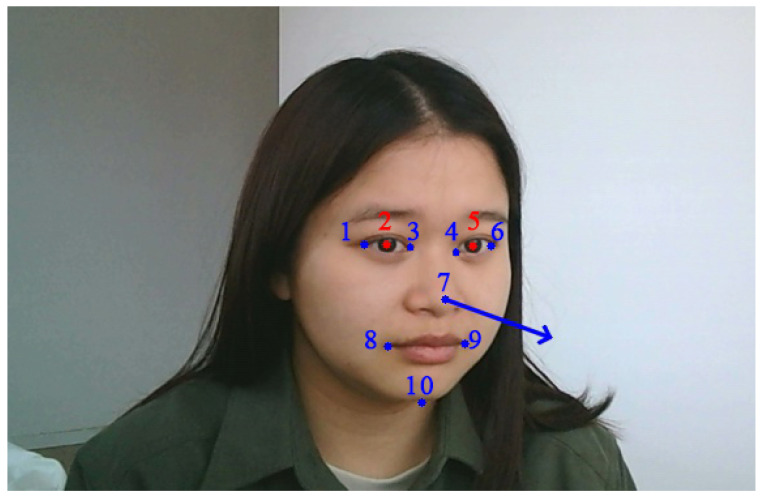
Feature extraction. The red dots 2 and 5 are the pupil centers, the numbers 1, 3, 4, and 6 show the points of the eye corner, and the numbers 1, 6, 7, 8, 9, and 10 are used to compute the head pose. The blue arrow line indicates the current head direction.

This section introduces the features required and the corresponding detection methods. The features contain the pupil center, head pose angle, and eye corner, as shown in Figure 2. First, the position of the pupil changes as the line of sight changes. Therefore, our method calculates the gaze point coordinates by detecting the change in the pupil center. Next, the head pose angle helps determine the head state and compute the reference point benchmark. Last, after the head motion stops, our method utilizes the eye-corner-related features to update the pupil center benchmark.

To obtain the above features, in this paper, we use the face mesh module [42] embedded in Mediapipe [10] to detect the feature points. Mediapipe is a highly optimized neural network framework whose efficiency and accuracy have been proven in many studies [43,44,45]. The face mesh module in Mediapipe is a facial landmark tracking solution introduced by Kartynnik et al. [42]. It outputs 468 facial feature points and 10 pupil feature points from the facial image. Figure 2 shows some of the detected feature points. Head pose estimation is usually referred to as a Perspective-n-Point problem [46]. Therefore, we use the solvePnP method from OpenCV with several facial points to calculate the head pose angle.

Since the accuracy of the solvePNP algorithm relies on camera parameters during the process, the camera should be calibrated beforehand to obtain these parameters. We employ the commonly used checkerboard image for camera calibration. The main procedure is as follows: First, capture 20 checkerboard images from varying directions and distances. Next, detect corner points in each image and map them to corresponding 3D points in the world coordinate system. Finally, utilize the function calibrateCamera provided in OpenCV to compute the internal parameter matrix, distortion coefficients, and other parameters.

Once the calibration process is completed, we can apply solvePnP to estimate head pose in real time. First, multiple facial feature points are extracted, including the nose tip, chin, left and right mouth corners, and the left and right outer eye corners. Then, these points with corresponding 3D coordinates are used to compute the rotation matrix and the transformation vector. Next, splice them together to obtain the head pose matrix. Finally, decompose this matrix to get the head pose components, i.e., pitch, yaw, and roll angles.

### 3.2. Calibration Phase

**Figure 3:**
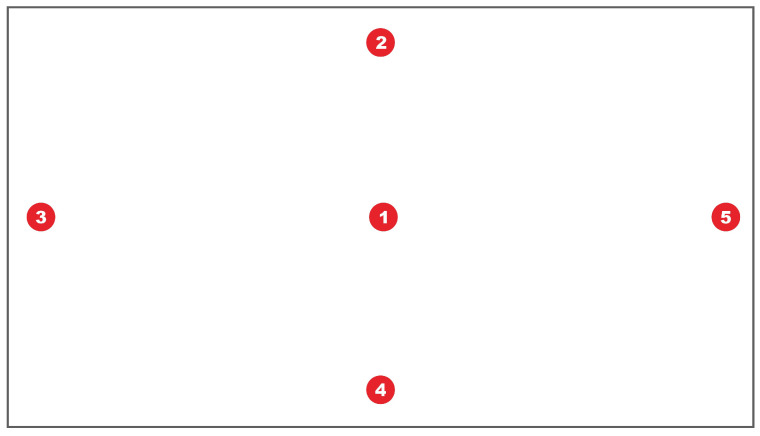
Calibration interface. The number in the dot represents the appearance order of the calibration point.

The purpose of calibration is to establish a projective relationship between the eye features and the gaze point coordinates. We use five points to conduct the calibration. The procedure of calibration is as follows: First, the middle area of the screen displays the image captured by the camera, with a pixel size of 640×480. Users need to place their heads within this range. Then, click the space bar on the keyboard to enter the calibration interface, as shown in Figure 3.

Five calibration points (one center point and four edge points) will appear in order. Users need to gaze at each one for about 2 s. Our method continuously detects the user’s head pose, pupil center, and eye corner during this period. At each calibration point, we save the mean value of each feature in the last 0.5 s for subsequent calculations. The features obtained in the first calibration point (screen center) are the reference benchmarks. In addition, the upper and lower points determine the scale coefficients of the Y-axis, and the left and right points compute the X-axis coefficients. Supposing that the coordinates of these four edge points are $C_{i}\left(x,y\right),i=2,\ldots ,5$, the pupil center coordinates of the left and right eyes when the user gazes at each point are $L_{i}\left(x,y\right)$ and $R_{i}\left(x,y\right),\left(i=2,\ldots ,5\right)$. Then, the scale coefficients Ls and Rs for the left and right eyes can be computed by the following equations.

(1) $$Ls(x,y)=(\frac{C_{5}\left(x\right)-C_{3}\left(x\right)}{L_{5}\left(x\right)-L_{3}\left(x\right)},\frac{C_{4}\left(y\right)-C_{2}\left(y\right)}{L_{4}\left(y\right)-L_{2}\left(y\right)})$$

(2) $$Rs(x,y)=(\frac{C_{5}\left(x\right)-C_{3}\left(x\right)}{R_{5}\left(x\right)-R_{3}\left(x\right)},\frac{C_{4}\left(y\right)-C_{2}\left(y\right)}{R_{4}\left(y\right)-R_{2}\left(y\right)})$$

To ensure that the system accurately predicts the gaze point coordinates, we ask the user to keep the head fixed during calibration. If the head poses at these five points changed beyond the threshold, the system will determine the calibration is unsuccessful, and the user needs to recalibrate.

In sum, after finishing calibration, we can obtain the scale coefficients of the left and right eyes (Ls and Rs) and several initial benchmark features, including the reference point (screen center), the eye corners, the pupil centers, and the head pose (pitch and yaw angles). For ease of understanding, we explain the term benchmark used in the paper. This study includes four benchmarks: the reference point, the pupil center, the eye corner, and the head pose. The reference point benchmark is the position where the head is pointing. The pupil center benchmark indicates the pupil center and eye corner coordinates when the user stares at the reference point. The eye corner and the head pose benchmarks denote the eye corner coordinates and the head pose angle at the first reference point, respectively. In these benchmarks, the former two change with the user’s head movement, while the rest remain fixed.

### 3.3. Eye-Tracking Phase

#### 3.3.1. Head State Detection

After calibration, the system starts tracking gaze points. For each subsequent frame, the first step is determining whether the head is moving. We divide the head states into head fixation and head motion. The former state means that the head remains fixed or moves slightly, and the system will further predict the gaze point coordinates, as described in Section 3.3.4. The latter state indicates that the head moves or rotates a wide range relative to the reference point benchmark, and the angular deviation between the current head pose angle and the previous exceeds the threshold. This study uses an empirical value of 2 degrees as the threshold to determine whether the head is moving. When the system determines that the user’s head is moving, the system further calculates the head-pointing coordinates and updates benchmark features, as described in Section 3.3.2 and Section 3.3.3, respectively, and then computes the gaze point with new benchmark features.

#### 3.3.2. Head-Pointing Computation

**Figure 4:**
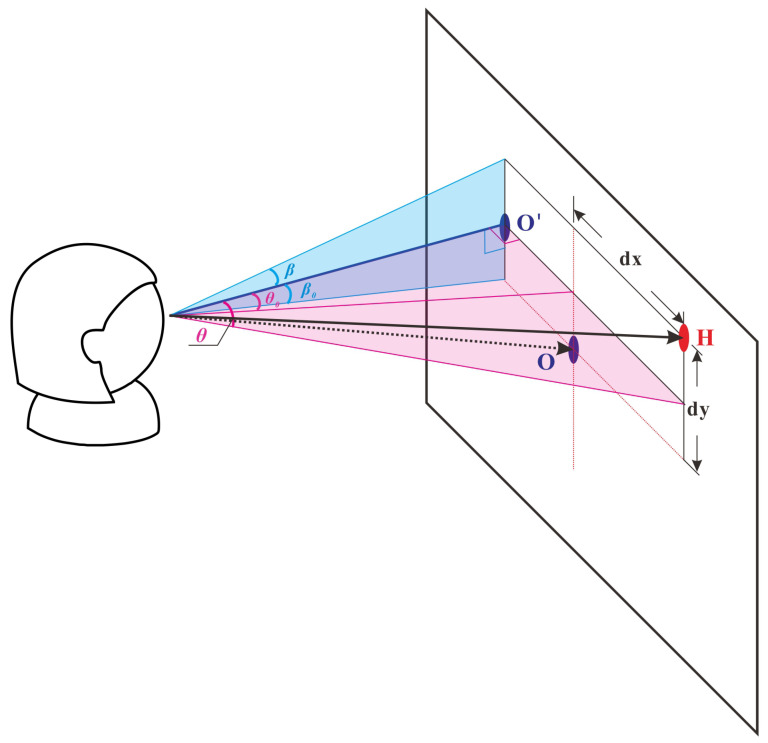
Head-pointing computation. The black dotted line denotes the head direction when gazing at the first point. The blue dot O in the screen center is the first calibration point, and $O^{\prime }$ indicates the projection position of the head on the screen. The black line is the current head direction. Point H is the head-pointing position to be calculated.

Figure 4 shows a schematic diagram of the head-pointing coordinate calculation. We do not require that the user exactly sit in the front center of the computer screen. They can sit in any position they feel comfortable, as long as their head and eyes are not out of the capture range of the camera. Therefore, during calibration, the user’s head direction may not be perpendicular to the screen. After the head moves, we calculate their head pose (yaw and pitch angles) in the current frame using the method introduced in Section 3.1. Then, we can compute the coordinates of head-pointing H(x,y) at the current pose using the following formulas.

(3) $$dx=d\times (tan\theta -tan\theta_{0})$$

(4) $$dy=d\times (tan\beta +tan\beta_{0})$$

(5) $$H\left(x,y\right)=\left(O_{x}+dx,O_{y}-dy\right)$$

where *d* represents the user-screen distance, and θ and β denote the yaw and pitch angles of the current frame, respectively. $\theta_{0}$ and $\beta_{0}$ are the yaw and pitch angles (head pose benchmark) at the first calibration point, and O(x,y) is the screen center coordinate.

#### 3.3.3. Benchmark Features Updating

When the head moves, the system has to update the reference point benchmark (head-pointing) and the corresponding pupil center benchmark. Intuitively, the coordinates of the facial feature points change after the head moves. We observe that the closer the feature points are, the more similar the distance they change. Since we aim to calculate the pupil center benchmark at the new head pose, we consider using the points near the pupil (inner and outer eye corners) to approximate the offset of the pupil center benchmark.

**Figure 5:**
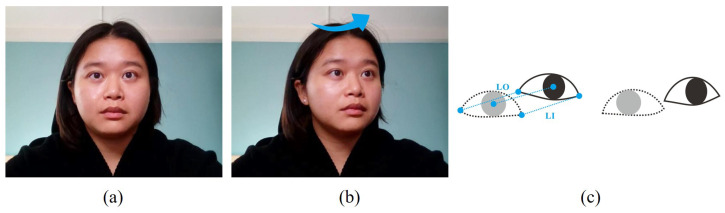
(**a**) Head fixation. (**b**) Head movement. The blue arrow indicates that the user rotates their head relative to the reference point benchmark. (**c**) Eye corner and pupil center vary after the head moves.

Suppose that the user moves their head relative to the reference point benchmark, as shown in Figure 5. We first calculate the motion distances of the eye corner. For the left eye, we denote the distance moved by the inner and outer corners of the eye as LI and LO, respectively, as shown in Figure 5c. Similarly, for the right eye, we mark the distances as RI and RO. Then, we take the mean distance moved of the inner and outer corners to approximate the offset of the pupil center benchmark for that eye. The new pupil center benchmark (Lc* and Rc*) can be obtained by the following formulas:

(6) $$Lc^{*}\left(x,y\right)=\left(Lc_{x}+\frac{LI_{x}+LO_{x}}{2},Lc_{y}+\frac{LI_{y}+LO_{y}}{2}\right)$$

(7) $$Rc^{*}\left(x,y\right)=\left(Rc_{x}+\frac{RI_{x}+RO_{x}}{2},Rc_{y}+\frac{RI_{y}+RO_{y}}{2}\right)$$

where Lc and Rc are the left and right pupil center coordinates at the first calibration point. Once the user’s head stops moving, we update the reference point benchmark with the current head-pointing coordinates computed by the Equation (5).

#### 3.3.4. Gaze Point Prediction

The method calculates the coordinates of the gaze point when the head keeps stationary or after updating benchmark features if the head is moving. First, we obtain the user’s pupil center at the current frame using the method introduced in Section 3.1 and compute its deviation (Lb and Rb) from the pupil center benchmark with the Equations (8) and (9).

(8) $$Lb=(L_{x}-Lc_{x}^{*},L_{y}-Lc_{y}^{*})$$

(9) $$Rb=(R_{x}-Rc_{x}^{*},R_{y}-Rc_{y}^{*})$$

where *L* and *R* separately represent the current left and right pupil centers. Then, the gaze point coordinates of the left and right eyes can be obtained using the following equations.

(10) $$LG\left(x,y\right)=\left(H_{x}+Lb_{x}\times Ls_{x},H_{y}+Lb_{y}\times Ls_{y}\right)$$

(11) $$RG\left(x,y\right)=\left(H_{x}+Rb_{x}\times Rs_{x},H_{y}+Rb_{y}\times Rs_{y}\right)$$

where *H* is the current reference point benchmark, and Ls and Rs are the scale coefficients obtained from calibration. Finally, we take the average coordinates of LG and RG as the user’s gaze point on the screen for the current moment.

## 4. Experiment

### 4.1. Participants

We invited 22 subjects (11 males) to participate in this experiment. Their ages ranged between 18 and 42. None of them has eye-related problems or irregular vision. The experiment was conducted in a quiet room with sufficient brightness. When the subjects arrived at the lab at the assigned time, the authors would thoroughly illustrate the experimental requests and contents to each one. Then, participants voluntarily signed an informed consent form, which included permission for video recording and subsequent analysis and use of their facial images. The experiment software functioned on a desktop PC with an Intel Core i7 CPU and an NVIDIA GeForce GTX 1080 GPU. The camera we used for image capture is a Logitech C270 webcam, which works at 30 frames per second with a maximum resolution of 720p. It utilizes a fixed focal length lens with an approximate focal length of 3.6 mm, and the diagonal field of view is about 60 degrees. The resolution of the screen is 1920×1080 pixels, and the distance between the user and the screen is around 50 cm.

The commonly used metric, visual angle error [47,48], was introduced to evaluate the accuracy of the proposed method. A lower error indicates better accuracy.

### 4.2. Task 1. Fixation Task

The first task required subjects to gaze sequentially at multiple dots on the screen. Each subject needed to complete the experiment with and without head motions. Additionally, since the accuracy of head pose could affect the precision of head-pointing coordinate and subsequent gaze point estimation when the head moves, this task also compares the accuracy of our method using Mediapipe [10] and two recent deep learning-based head pose detectors, Lightweight [49] and 6DRepNet [50], in head motion. Thus, subjects were required to complete a total of four trials.

#### 4.2.1. Experimental Procedure

**Figure 6:**
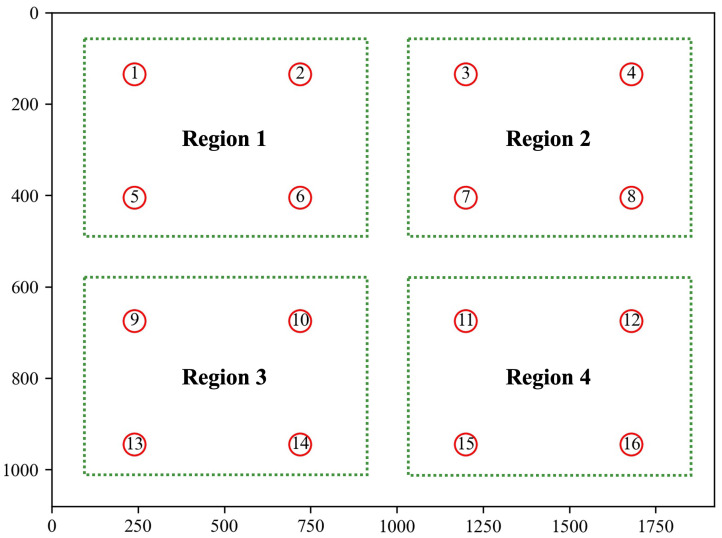
Experimental interface. The x- and y-axes values refer to the pixel value, and the numbers indicate the order in which each test point is displayed.

When calibration is complete, the software switches to the test interface in which 16 red dots will appear from left to right and top to bottom, as shown in Figure 6. Each dot appeared for 3 s. Subjects were required to gaze at the dot until it disappeared. In the head fixation mode, we asked the subjects to keep their head as still as possible during the test. In the head motion mode, the subject can freely rotate the head to a position where the eyes can more comfortably focus on the test dot. However, the head motion should be limited to a range to guarantee the head-pointing coordinates fall within the screen. The angular ranges of yaw and pitch are ±18° and ±10°, respectively. These ranges were determined by the subjects’ seating positions and the screen dimensions. Once the head motions exceeded the range, a prompt would appear on the screen, and the subjects needed to rotate their head as required. The predicted gaze points of the subject at each test dot are recorded. After completing all 16 points, the visual angle error between the estimated gaze points and actual test points would be automatically computed.

#### 4.2.2. Experimental Results

**Figure 7:**
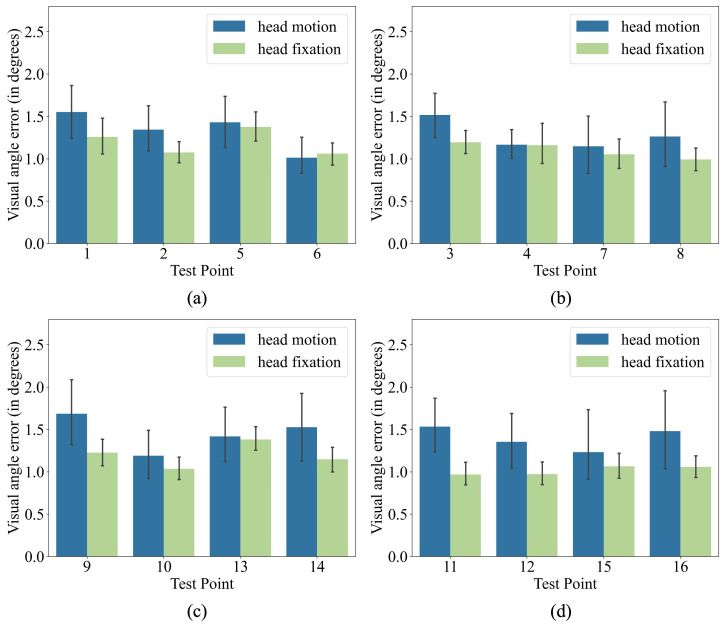
(**a**–**d**) separately show the comparison results of accuracy in each region.

The test interface was divided into four equal-sized regions (see Figure 6) with four test dots each to compare the prediction accuracy of our method under head fixation and head motion. Figure 7 shows the average prediction errors in each region. The head fixation mode has lower errors than the head motion mode in all four regions. It is expected because most current eye-tracking methods have much higher errors under head movement than under head fixation. Additionally, our method has a visual angle error below 1.5 degrees at more than half of the test points in head motion mode, and the prediction errors at most of the test points are higher than that in head fixation by less than 0.5 degrees. In addition, the performance of our method in head motion mode is close to that in head fixation at three test points (No. 4, 6, and 13). The above results indicate that our method achieves an acceptable accuracy with head motion.

**Figure 8:**
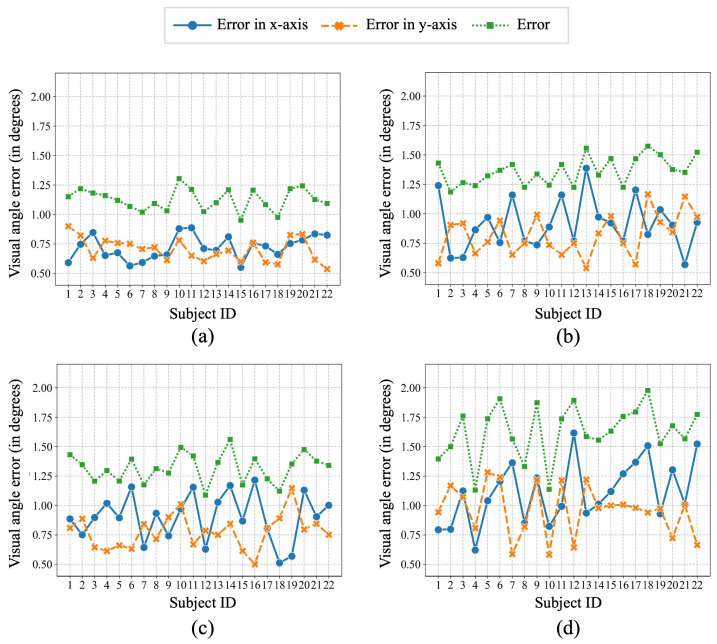
Visual angle error of each subject using the proposed method under head fixation (**a**) and head motion (**b**). The X-axis is the number of subjects, and the Y-axis displays the error (in degrees). Blue and orange dots separately indicate errors in the x- and y-axes, while green dots reflect the overall error. In addition, (**c**,**d**) show the error of each subject using different head pose detectors: 6DRepNet [50] and Lightweight [49] under head motion.

Next, we analyze the results for each subject under head fixation and head motion. Figure 8a and Figure 8b separately show the gaze accuracy of our method under head fixation and head motion. The errors in x- and y-axes under head fixation were less than 1 degree and fluctuated smaller than those under head motion. In addition, most subjects’ errors under head fixation were slightly lower than under head motion. Specifically, the errors under head fixation were between 1 and 1.25 degrees, while between 1.25 and 1.5 degrees under head motion.

Then, we compare the accuracy of our methods using different head pose detectors. As can be seen from Figure 8b–d, the gaze errors of 6DRepNet and Mediapipe were more stable than those of Lightweight. The average error was lowest with 6DRepNet (1.32 degrees), followed by Mediapipe (1.37 degrees) and Lightweight (1.63 degrees). 6DRepNet also achieved the lowest average errors in the x- and y-axes. Although the error of Mediapipe is slightly higher than that of 6DRepNet, it requires fewer hardware resources and could run smoothly. Therefore, for the remainder of the experiment, Mediapipe was employed exclusively for head pose detection.

Table 1 compares the accuracy of our method with existing camera-based eye-tracking methods with and without head motion. Our method achieves a average error of 1.13 and 1.37 degrees in head fixation and head motion modes, respectively. Under head fixation, we can observe that our method achieves the minimum error over other methods. Compared with Cheung et al. [51], which ranks second among all, our method improved the accuracy by about 0.15 degrees. Under head motion, our method outperforms all the methods except Guo et al. [9] and Liu et al. [40]. The error of our method is higher than theirs by about 0.17 and 0.04 degrees, respectively. However, the experiment of Guo et al. [9] was conducted with slight head movements, and the authors reported a poor visual angle error beyond 5 degrees under free head movements. The method of Liu et al. [40] required two additional infrared light sources, which might increased the economic costs and restricted the experimental environment.

### 4.3. Task 2. Smooth Pursuit

**Figure 9:**
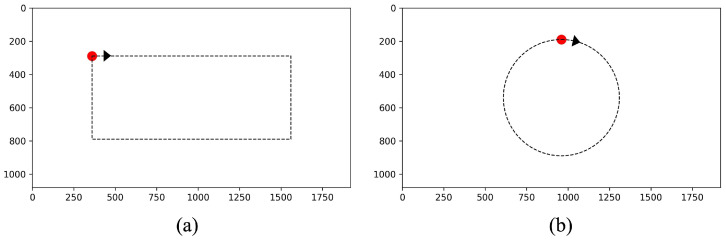
Smooth pursuit task interface. (**a**) Rectangular motion trajectory. (**b**) Circular motion trajectory. The red dot indicates the target that the user should gaze at, and the black arrowhead is the direction the target moves.

This section employs a smooth pursuit experimental paradigm to quantitatively evaluate the accuracy when participants gaze at a moving target. The experimental protocol requires participants to maintain steady gazes on the moving target throughout the trials under two predetermined motion trajectories (rectangular and circular). Figure 9 shows the interface of this task.

#### 4.3.1. Experimental Procedure

The procedure of this experiment was as follows: First, participants selected head mode between head fixation and head motion, adjusted their seat to the testing position (50 cm from the screen center), and completed the 5-point calibration procedure. Upon readiness, the screen switched to the rectangular trajectory testing interface (Figure 9a), where a red circular target (diameter: 40 pixels) appeared at the upper-left screen quadrant. Participants were instructed to fixate on the target centroid and press the spacebar to initiate its clockwise rectangular motion at 120 pixels/s. Continuous gazing was required until the target returned to the starting position. After completing trials under the current head mode, participants repeated the above procedure with the alternate mode. After the rectangular trajectory testing, a 1–2 min rest interval was enforced to alleviate ocular muscle fatigue. The protocol then progressed to the circular trajectory phase (Figure 9b), where an identical target appeared at the upper-central screen region. The target executed a clockwise circular motion at 15°/s. The radius of the circle is 350 pixels. Participants performed one trial per head mode. Crucially, both trajectories concealed path information, displaying only real-time target positions. Throughout the experiment, gaze coordinates were recorded at a 30Hz sampling rate. Tracking precision was quantified using the mean visual angle error between gaze points and target centroids during motion cycles.

#### 4.3.2. Experimental Results

Table 2 presents the mean gaze accuracy and variance of all participants across distinct trajectories and head movement conditions. First, we compared the accuracy of different head movement modes under the same trajectory. *T*-test results showed that under the rectangle trajectory, the accuracy of the head fixation mode was significantly higher than that of the head motion mode by about 0.38° (t = 9.48, *p* < 0.001). Under the circular trajectory, the head-fixed mode accuracy advantage was even more significant and was about 0.41° higher (t = 14.18, *p* < 0.001). Next, the accuracy between two trajectories in the same head movement mode was compared. *T*-test results showed that the rectangle trajectory accuracy was significantly lower than the circular trajectory by about 0.11° under the head fixation mode (t = −2.335, *p* = 0.03). In addition, there was no significant difference under the head motion mode (t = 1.95, *p* > 0.05).

Table 3 shows the root-mean-square error (RMSE) of pixels for different trajectories and head movement modes. It can be seen that the x-axis RMSE of the circular trajectory is lower than that of the rectangular trajectory by about 5.8 and 8.6 pixels for the head fixation and head motion modes, respectively. In addition, the y-axis RMSE stays at an approximate level with that of the rectangular.

In sum, in both head modes, the accuracy of our method in the rectangle trajectory is slightly lower than that of the circular. For both trajectories, the average accuracies of our method are 1.40° and 1.79° under head fixation and head motion, respectively. Although the accuracies decreased by about 0.27° and 0.42° compared with the results obtained from the fixation-point experiment, they are still in the acceptable range (<2°). The experimental results demonstrate that the proposed method could maintain an effective tracking capability in complex motion scenarios.

## 5. Case Study

This section applies the proposed method to analyze user experience within actual game scenarios through a case study. Understanding players’ game experience is one of the primary objectives of game design [57], since it could influence their attitudes toward the game and their willingness to continue playing. By conducting user experience research, designers can better identify players’ needs and preferences, thereby optimizing game design and attracting more users.

To analyze visual behavior, it is necessary to convert gaze point coordinates into eye movement metrics, such as the fixation count and heatmaps. The methods used in this paper are briefly described below. First, we utilize the Velocity-Threshold Identification (IVT) algorithm [58] to classify the predicted eye gaze coordinates into fixation and saccade. Fixation and saccade are the two most common eye movement behaviors during interaction. Among them, fixation implies that the user’s eye stays at a specific place or target over a certain period to observe it more clearly, while saccade represents the procedure of rapid eye movements between two neighboring fixation points [59]. We use the IVT algorithm employed in the Tobii eye-tracker [60] to classify the gaze coordinates into fixation and saccade. The basic idea of IVT is to classify eye movements by calculating the speed of eye movement in the horizontal and vertical directions. In addition, the gaze heatmap with various colors is calculated from all gaze points and provides a clear reflection of the gaze area. The red color represents where the user pays the most attention, followed by yellow, green, and blue [4].

This study selects the classic puzzle game Zuma as experimental material, aiming to explore differences in game experience among users with varying experience across different game modes.

### 5.1. Participants

The study involved a total of 16 participants (8 males), a sample size adequate for the initial demonstration and proof-of-concept purposes of this work. Among them, 8 were experienced players (averaging over 2 h of daily gameplay), while the rest were inexperienced (averaging less than 2 h of daily gameplay). Experience may influence participants’ gaze behavior and preferences, so this study incorporated it as a key grouping variable in the experimental design. The average age was 35.6 years, with a standard deviation of 11.7. Participants included students, teachers, engineers, and others. All participants were right-handed and in good physical health. Some participants wore prescription glasses with corrected vision. When they arrived at the lab at the assigned time, the authors would thoroughly illustrate the experimental requests and contents to each one. Then, participants signed the consent and completed the experiment in a quiet and well-lit room. Upon completion, they would receive a gift worth 10 yuan as a token of appreciation.

### 5.2. Experimental Material

**Figure 10:**
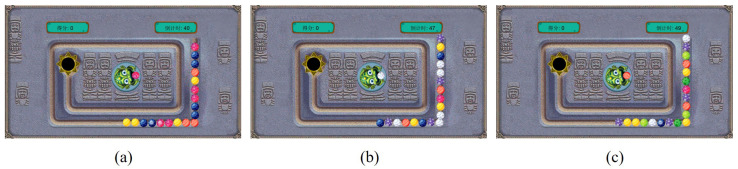
(**a**–**c**) separately show the experimental interface of each game mode. The Chinese characters on the left and right of the interface represent the score and countdown, respectively. Besides, the number of bead colors in the three modes is 4, 6, and 8, respectively.

We used the popular Zuma game as the experimental material. The rules are as follows: During gameplay, a string of multicolored beads rolls along a track toward the black hole. Players control the head direction of the toad within the game interface by moving the mouse, then click the left mouse button to shoot the colored bead from the toad’s mouth, preventing these beads from entering the black hole. The color of the bead in the toad’s mouth appears randomly each time. When players shoot it at the track bead string to connect three or more beads with the same color, that string of matching beads immediately disappears. The number of disappearing beads equals the points the user got. Subsequently, the bead strings in front of the disappearing position will wait for the strings behind them to move closer before advancing together. If the leftmost bead reaches the black hole within the allotted time, the game ends early. Building upon the original game, this study designed and developed a version with three difficulty modes using Python 3.9.15. The interfaces and settings for different modes are illustrated in Figure 10 and listed in Table 4, respectively.

As shown in Table 4, the three modes differ in the number of bead colors and speed. Figure 10a depicts mode 1, which is configured as the simplest mode, featuring four colors and the slowest ball speed. Figure 10b shows mode 2, featuring a bead string with six colors and a ball speed slightly faster than mode 1. Figure 4c depicts mode 3, which has the most colors in the bead string and moves faster than mode 2. The game interface displays the score and the countdown timer on the top left and right, respectively. Each mode lasts for one minute.

### 5.3. Experimental Procedure

The main procedure of the experiment is as follows: after introducing the game content and rules to participants, they first play the game one or two times to familiarize themselves with the game. The formal experiment then commences. Participants randomly select a game mode and proceed to execute the calibration process. After calibration is complete, the game starts. Participants control the head direction of the toad by moving the mouse and click the left mouse button to shoot the bead string. To achieve a higher game score, they must aim the bead as accurately as possible at the bead strings of the same color on the track. During game, the experimental software tracks the user’s gaze points in real time. When the game ends, the software automatically stops recording data and saves the game score. Then, participants need to rate the difficulty level and preference for the game on two 5-point Likert scales. A score of 1 corresponds to “very easy” or “strongly disliked”, while a score of 5 indicates “very difficult” or “strongly liked”. After completing the questionnaire, participants rest for 1 to 2 min, select the next mode, and repeat the above steps. Upon conducting all three modes, the experiment ends, and the entire experiment takes approximately 10 min.

### 5.4. Experimental Results

#### 5.4.1. Results of Subjective Experiences and Game Score

Table 5 shows the mean subjective difficulty, preference, and game score for players with varying experience under different game modes. As expected, perceived difficulty increased with the number of bead colors and speed. Notably, while experienced players reported lower difficulty in Modes 1 and 2, they assigned a higher difficulty score to Mode 3 than inexperienced players. In terms of preference, inexperienced players favored the medium-difficulty Mode 2, while experienced players preferred the most challenging Mode 3. As game difficulty increased, the preference ratings of inexperienced players showed a trend of rise first and then fall. Differently, experienced players tend to give higher ratings. It suggests that they are more motivated by high challenges. Moreover, the overall decline in game score with increasing difficulty confirms that the three game modes effectively represent distinct difficulty levels.

#### 5.4.2. Results of Eye-Tracking Data

**Figure 11:**
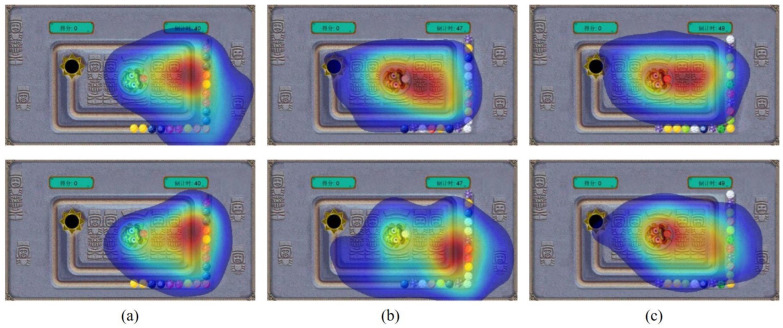
(**a**–**c**) separately show the gaze heatmap of each game mode. The first and second rows present the results for inexperienced and experienced players, respectively. The Chinese characters on the left and right of the interface represent the score and countdown, respectively. Besides, the red color indicates the areas where users are most focused.

Figure 11 displays the gaze heatmaps for player groups with varying levels of experience across three different game modes. Subfigures a–c correspond to Mode 1, Mode 2, and Mode 3, respectively. As shown in the figure, in Mode 1, the gaze heatmaps were similar, with both groups focusing on the right side of the interface, afforded by the slow bead speed. In Mode 2, the visual attention diverged: inexperienced players shifted their focus to the near center, indicating they were tracking beads that had entered the inner track, while experienced players maintained focus on the lower-right corner, suggesting superior ability and efficiency in clearing beads. In Mode 3, the high difficulty forced both groups’ focus to narrow towards the center, as beads rapidly accumulated on the inner tracks. Additionally, it can be observed that the differences in heatmaps between Mode 2 and Mode 3 are relatively minor for inexperienced players, implying that the difference in difficulty between these two modes has a limited impact on their performance.

Table 6 summarizes the fixation count for players with different experience across different modes. As shown in the table, the fixation count for inexperienced players decreases steadily as game difficulty increases, indicating they reduced their shooting frequency to ensure accuracy. In contrast, experienced players maintained a consistent fixation count, demonstrating greater composure and a stable operational rhythm despite the increased difficulty.

#### 5.4.3. Analysis of Subjective Experiences and Objective Eye-Tracking Results

By analyzing the results of subjective experiences with eye-tracking data, it can be observed that there is a close correspondence between players’ subjective perceptions and their visual behaviors. For inexperienced players, the subjective increase in difficulty and the initially climbing and then declining of their preference were mirrored by a contraction of their visual focus and a reduction in fixation points. These implied that inexperienced players were forced to reduce shooting frequency and adopt a more conservative fixation strategy under high difficulty, which was consistent with the significant drop in scores and diminished preference. On the contrary, while experienced players assigned higher difficulty ratings to Mode 3, they demonstrated greater preference and consistently higher game scores. Objectively, eye-tracking data also revealed that the fixation counts remained largely consistent across all modes. Specifically, the sustained focus on the bottom-right corner (where the beads fall vertically) in Mode 2 indicates stronger predictive skills and visual management. This ability to maintain efficient visual strategies under demanding conditions explains their sustained performance and preference for challenging modes.

In summary, this experiment investigated how different game modes affect the user experience of players with varying experience. The findings provide crucial insights into the interactive relationship between game difficulty, player experience, and visual behavior. In addition, it also confirms the effectiveness of eye-tracking in providing objective insights into user experience that complement traditional subjective measures.

## 6. Discussion

### 6.1. Comparison Against CNN Models

Currently, modern CNN-based approaches are becoming increasingly common in the field of gaze estimation, such as MPIIGAZE [61], GAZE360 [62], ETHXGaze [63], and FAZE [64]. Unlike gaze point prediction methods, these techniques typically estimate 3D gaze directions (i.e., pitch, yaw, and roll) through complicated CNN models instead of 2D gaze points. Therefore, to derive gaze points, the calibration operation is generally required to establish the projection model from gaze direction to gaze point. Table 7 lists the accuracy and frame rate (FPS) of several CNN-based gaze point estimation methods: MPIIGaze [61], ETH-XGaze [63], and FAZE [64]. From the table, we can see that FAZE obtained the highest accuracy, followed by ETH-XGaze, and MPIIGaze was the last. However, a better performance comes with higher computational efficiency. While FAZE achieves the highest accuracy (2.44 degrees), its frame rate is only approximately 1 Hz, making it hard to apply in scenarios with high real-time requirements.

In contrast, the proposed method employs a more efficient MobileNet-based MediaPipe solution to extract the pupil center and further compute head pose, head-pointing coordinates, and gaze point coordinates. The workflow achieves real-time operation using only the CPU with a cost of 14.2 ms (about 70 HZ) for each frame. Furthermore, this method also demonstrates excellent accuracy under head movements. In summary, despite its relatively simple architecture, the proposed method outperforms current CNN-based gaze estimation approaches in both computational efficiency and prediction accuracy.

### 6.2. Comparison Against Professional Eye Trackers

We compare our method against professional eye-trackers from two aspects: gaze accuracy and cost.

In terms of gaze accuracy, the proposed method still lags behind those professional eye trackers. Table 8 shows the accuracy of some commercial eye-trackers, such as Tobii 3, Pupil Labs Glasses, and EyeLink 1000, their error ranges typically fall between 0.5 and 0.8 degrees. These eye trackers are generally equipped with high-precision infrared sensors, capable of capturing ocular dynamics with greater precision, thus obtaining satisfactory accuracy.

Then, we compare our method with professional eye-tracking devices based on cost. This study employs ordinary cameras to capture users’ gaze points, resulting in low hardware costs, generally less than 200 RMB. Moreover, our approach also offers advantages in terms of deployment and operation. In contrast, most professional eye trackers rely on specialized hardware, significantly increasing expenses. These devices typically cost over 1000 dollars. Some high-end devices even reach tens of thousands of dollars. Furthermore, these devices are relatively complex to operate, often requiring specialized training before users can master their use.

In summary, we must acknowledge that this study has a significant gap compared to professional eye trackers in terms of accuracy. However, our method also offers the following advantages: (1) Low economic cost: Only a standard camera is required to achieve head-motion-adaptive gaze point estimation, eliminating the need for additional hardware; (2) Easy deployment: Can be rapidly deployed and used across various desktop environments; (3) Remote testing capability: No dedicated laboratory setup is required; participants can engage in eye-tracking experiments using their personal computers; (4) Efficient data collection: Multiple participants can be recruited simultaneously for concurrent eye-tracking experiments, significantly reducing data collection time and accelerating experimental progress.

### 6.3. Limitations

Compared with Falch et al. [39], our method shows superior accuracy when the head is kept fixed. However, the proposed method has limitations in handling large head movements, which stem from inherent characteristics of the approach. We achieve accurate gaze point estimation during head rotation by calculating the orientation coordinates after head rotation and dynamically updating the pupil center benchmark. Experimental results demonstrate that within the defined range of head movements, our method exhibits robust performance and promising accuracy. Nevertheless, when head motion exceeds the expected range, the reliability of facial feature detection diminishes. This degradation affects the accuracy of benchmarks such as eye corner, head pose, and pupil center, ultimately leading to increased gaze point estimation errors. On the contrary, Falch et al. [39] focus on building a robust gaze point estimation under large head movements. They incorporate Structure from Motion into the gaze point regression model, demonstrating satisfactory performance across a wide range of head motions. However, in most research scenarios, head rotation is typically restricted to a limited scope. Therefore, our method maintains stable performance under these conditions. In the future, we will also consider integrating the approach from Falch et al. [39] to expand the applicability and robustness of our method during extensive head movements.

Second, although this paper has validated the effectiveness of the proposed eye-tracking method in user experience research, the limited sample size restricts the generalizability of the findings. Future research will validate the effectiveness in studies with adequate sample sizes. Additionally, this study analyzes user experience based on the fixation count and gaze heatmaps. Future research may incorporate additional metrics such as first fixation, dwell time, and browsing paths to comprehensively reveal users’ visual cognitive patterns.

Third, the practical applicability of this method in other scenarios requires further investigation. Applying camera-based eye-tracking to computer games for interaction also presents a promising avenue to evaluate its performance in real-world settings. In future work, we will investigate the feasibility of using eye-tracking as a modality for game-based interaction.

## 7. Conclusions

The primary contribution of this paper is to propose an eye-tracking method that is robust against head movements. Our method detects the head motion state according to the head pose. When the head is moving, the reference point and pupil center benchmarks are updated immediately. Afterward, the method predicts gaze point coordinates in real-time by calculating the deviation of the pupil center from the new pupil center benchmark. The accuracy of the proposed method was validated through two tasks: fixation and smooth pursuit. The experimental results demonstrated the effectiveness of our method. Additionally, we also employ our method to analyze visual behavior and user experience when playing games. The experimental results indicated that eye-tracking metrics obtained by our method can help explain users’ subjective experiences.

## Figures and Tables

**Table 1:** Visual angle error (in degrees) comparison with other camera-based eye-tracking methods.

Approaches	Head Fixation	Head Motion
Sasaki et al. [48]	1.46	-
Ansari et al. [52]	1.98	-
Koshikawa et al. [53]	2.1	-
Hu et al. [47]	-	2.67
Guo et al. [9]	-	1.2
Li et al. [54]	-	2.0
Lu et al. [55]	-	2.5
Cheung et al. [51]	1.28	2.27
Banks et al. [41]	-	2.67
Falch et al. [39]	3.2	-
Liu et al. [40]	-	1.33
Zhou et al. [56]	-	1.99
Proposed	1.13	1.37

**Table 2:** Gaze accuracy of smooth pursuit task.

Mode	Rectangular	Circluar
Head fixation	1.45 (0.15)	1.34 (0.16)
Head motion	1.83 (0.14)	1.74 (0.14)

**Table 3:** RMSE of two trajectories on the x- and y-axes.

Mode	Rectangular		Circluar	
	rmse_x	rmse_y	rmse_x	rmse_y
Head fixation	56.1 (9.0)	57.8 (7.6)	50.3 (9.6)	56.6 (7.6)
Head motion	77.4 (9.0)	65.1 (11.3)	68.8 (7.3)	67.4 (11.6)

**Table 4:** Game settings.

Game Mode	Number of the Bead Colors	Speed of the Bead
Mode 1	4	1.2
Mode 2	6	1.5
Mode 3	8	1.6

**Table 5:** Mean subjective difficulty, preference, and game score for players with varying experience under different game modes.

Metrics	User Type	Mode 1	Mode 2	Mode 3
Difficulty	inexperienced	1.75	3.13	4.13
	experienced	1.63	2.63	4.50
Preference	inexperienced	3.25	3.5	3.0
	experienced	3.25	3.38	3.63
Game score	inexperienced	50.71	29.25	21.75
	experienced	58.63	41.75	30.50

**Table 6:** Fixation count for players with varying experience across different modes.

Metric	User Type	Mode 1	Mode 2	Mode 3
fixation count	inexperienced	91.63	83.88	76.13
	experienced	77.50	77.63	74.25

**Table 7:** Visual angle error (in degree) and FPS of some CNN-based eye-tracking methods.

Methods	Accuracy	FPS (HZ)
MPIIGAZE [61]	3.70	12
ETHXGaze [63]	3.40	4
FAZE [64]	2.44	1

The above results were obtained from Saxena et al. [65].

**Table 8:** Visual angle error (in degree) of some commercial eye-trackers.

Reference	Devices	Accuracy
Onkhar et al. [66]	Tobii 3	1.60
Housholder et al. [67]	Tobii 5	0.74
Ehinger et al. [68]	EyeLink 1000	0.57
Ehinger et al. [68]	Pupil Labs glasses	0.82

## Data Availability

The code that support the findings of this study are available at https://github.com/mint-deeplearning/Eyetracking (accessed on 20 November 2025). The data presented in this study are available upon request from the corresponding author. The data are not publicly available due to restrictions, e.g., privacy or ethical.
